# The effect of antibiotics on the periodontal treatment of diabetic patients with periodontitis: A systematic review and meta-analysis

**DOI:** 10.3389/fphar.2023.1013958

**Published:** 2023-01-25

**Authors:** Ziwei Tang, Qi Fan, Qingsong Jiang, Xiaolong Li, Yan Wang, Hu Long, Wenli Lai, Fan Jian

**Affiliations:** State Key Laboratory of Oral Diseases, Department of Orthodontics, West China Hospital of Stomatology, Sichuan University, Chengdu, China

**Keywords:** diabetes, periodontitis, scaling and root planning, antibiotics, systematic review

## Abstract

**Background:** The aim of this meta-analysis was to compare the effects of periodontal treatment with or without adjunctive antibiotic on periodontal status and blood glucose level in diabetic patients with periodontitis.

**Methods:** A search using electronic database (MEDLINE, EMBASE, and Cochrane Central Register of Controlled Trials) and a manual search were performed up to July 2022. Eligible 13 RCTs were included according to inclusion and exclusion criteria. Reviewers independently performed data screening, data selection, data extraction, and risk of bias. Quality assessment was performed according to the Cochrane Handbook for Systematic Reviews of Interventions. Weighted mean differences and 95% confidence intervals (CIs) for continuous outcomes were calculated using random or fixed-effects models. This review is registered in the PROSPERO database (CRD42022347803).

**Results:** Of the 13 included articles, eight were on the use of systemic antibiotics and five on topical antibiotics. The results showed statistically significant improvement in periodontal status (probing depth, clinical attachment loss and bleeding on probing) at 6 months with systematic antibiotics use (PD-6M *p* = 0.04, BOP-6M *p* < 0.0001, CAL-6M *p* = 0.002). The improvement in PD with topical antibiotics was statistically significant at 1 month (*p* = 0.0006). However, there was no statistically significant improvement in periodontal status at 3 months with adjuvant systemic antibiotics.

**Conclusion:** Antibiotics can improve the periodontal condition of diabetic patients with periodontitis to a certain extent. In clinical practice, it is necessary to comprehensively consider the balance of benefits and risks before deciding whether to use antibiotics.

**Systematic Review Registration:** Identifier CRD42022347803, https://www.crd.york.ac.uk/PROSPERO/.

## Introduction

Diabetes mellitus is a chronic systemic disease caused by loss of functional pancreatic beta cells which often requires long-term treatment to control the blood sugar levels (Oppermann et al., 2012). In Type I diabetes mellitus (T1DM) autoimmune cells attack pancreatic islet and thus leading to damage of pancreatic beta cells. The etiology of type 2 diabetes mellitus (T2DM) is complex and mostly related to genetics, smoking, age, etc. (Boldison & Wong, 2016). Periodontitis is mainly an infectious disease which initiated by the biofilm on the tooth surface, followed by host immune response producing cytokines, pro-inflammatory mediators, and finally resulted in reorption of alveolare bone and loss of attachment (Oppermann et al., 2012; Preshaw et al., 2012).

Diabetes can lead to dysregulation of host inflammatory responses and increased destruction in tissues and dysfunction in organs. Relationship between periodontitis and diabetes has risen increasing interest recent years, however the underlying mechanism remains unclear. Many studies considered diabetes as a risk factor for periodontitis. It is found that patients with diabetics had worse periodontal conditions compared with those who without (Faria-Almeida et al., 2006; Lalla et al., 2006; Lalla et al., 2007; Fernandes et al., 2009; Santos et al., 2009; Bandyopadhyay et al., 2010; Rovai et al., 2016), and patients with diabetics were 3–4 times more likely to develop periodontitis than normoglycemic patients (Graves et al., 2020). The molecular and cellular mechanisms underlying the relationship between periodontitis and diabetes have been extensively studied. Increasing inflammatory factors were observed in the periodontal tissue, and resulting in more severe periodontal tissue destruction (Polak and Shapira, 2018). Elevated levels of inflammatory factors such as IL-1β, IL-6, and tumor necrosis factor (TNF)-α was found in the gingival sulcus and saliva. Increased RANKL/osteoprotegerin ratio as well as negatively affected neutrophil function were also been noticed (Taylor et al., 2013). All those findings offered a perspective that diabetes breaks periodontal homeostasis and leads to periodontal tissue destruction aggravation *via* inflammatory changes.

For most periodontitis patients control of periodontal condition is mainly based on periodontal treatment including periodontal scaling and root planning (SRP). Local or systemic drug adjuvant treatment is rarely needed. For patients with severe periodontal destruction or immune irregulating diseases such as diabetes, periodontal treatment in combination with systemic antibiotics is used to control periodontal infections, which can rapidly suppress target microbial species and accelerate the establishment of a host-compatible microbiota (Feres et al., 2015; Mainas et al., 2022). Topical application of metronidazole (Klinge et al., 1992; Lie et al., 1998), chlorhexidine (Pietruska et al., 2006) and some non-traditional antibiotics such as cashew gum (Ferreira-Fernandes et al., 2019) and salvadora persica gel (Niazi et al., 2020) have been found effective clinically in common periodontitis patients. Topical application of antibiotics, such as minocycline ointment, in patients with deep periodontal pockets has also been proved as a clinically effective option (Bharti et al., 2013). However, in the periodontal control of patients with systemic diseases such as diabetes, whether the use of antibiotics can improve the periodontal condition has been controversial. A systematic review by Maria et al. found that the combination of amoxicillin and metronidazole was more effective in improving periodontal probing depth (PD) (Souto et al., 2018). A more recent systematic review by Kenneth et al. found that systemic administration of doxycycline (Doxy) did not significantly improve clinical attachment levels in the periodontium, nor did it improve HbA1c levels (Yap and Pulikkotil, 2019). While in topical administration, Rovai et al. suggested that topical antibiotics can play a role in the improvement of probing depth (PD) and clinical attachment loss (CAL). In addition, some studies have found that periodontal therapy may have a positive effect on patients’ glycemic control (Lalla and Papapanou, 2011; Corbella et al., 2013; Wang et al., 2014). However, a 2017 meta-analysis reported no statistically significant improvement in HbA1c with adjunctive use of systemic antibiotics in periodontal therapy in patients with diabetes (Lira Junior et al., 2017).

Scaling and root planning is a commonly used clinical method for mechanical removal of plaque and calculus in patients with periodontitis. In some deep periodontal pockets, especially sites with PD > 5 mm, calculus cannot be completely removed (Deas et al., 2016). Persistence of inflammation may lead to local microbial escape from host immune defenses, resulting in persistent loss of attachment (Caton et al., 2000; Ehmke et al., 2005). However, the depth of probing was decreased, and the level of clinical attachment was increased after SRP in the vast majority of deep periodontal pockets. Moreover, studies have found that SRP is beneficial for reducing metabolism and systemic inflammation in T2MD patients (Baeza et al., 2020). On this basis, antibiotics can assist SRP for better periodontal treatment in diabetic patients (Santos et al., 2015; Grellmann et al., 2016).

There have been few systematic reviews of periodontal treatment in diabetic patients with periodontitis in recent years. Clinical trials included in the previous systematic review were not updated to the latest. Our study focuses on the most recent clinical trials and hopes to provide some ideas on whether it is effective to use antibiotics either systematically or topically while controlling the local periodontal condition with SRP in diabetic patients with periodontitis.

## Materials and methods

### Protocol

This article provides a systematic review of clinical trials. These studies investigated the effect of antibiotic use on the periodontal status of diabetic patients with periodontitis. This review report is based on PRISMA guideline (Moher et al., 2009) and is registered in the PROSPERO database (CDR42018103828).

### Research question

This systematic review highlights the following issues, mainly based on the PICOS principle: What is the effect of antibiotics as adjunctive therapy to periodontal therapy (I) versus no antibiotics (C) on periodontal status and glycated hemoglobin levels (O) in diabetic patients (P) with periodontitis in a randomized controlled trial?

### Study search strategy and study inclusion

A comprehensive review of literature was performed using search strategy developed for MEDLINE and revised for other individual databases including PubMed database, EMBASE database and Cochrane Central Register of Controlled Trials. The electronic search used different combinations with diabetes and periodontal disease as keywords. A combination of MesH terms and keywords is also used to identified the relevant clinical trials. Conference proceedings and abstracts in dentistry were searched for possible grey literatures. The reference lists of included clinical trials were also assessed to identify any additional studies. Study screening were done independently by two reviewers (ZT and FJ) following the inclusion and exclusion criteria listed below. Differences between reviewers were resolved by discussion with a third reviewer (WL).

#### Inclusion criteria

(1) Randomized controlled trials (RCT).
(2) Patients diagnosed with periodontitis and diabetes (T2DM or T1DM and T2DM).
(3) Studies evaluating the clinical effects of all antibiotics and no antibiotics.
(4) Studies reporting one or more clinical periodontal parameters as outcomes, including PD or CAL, bleeding on probing (BOP%), and changes in HbA1c before and after treatment.
(5) Research published in English.

#### Exclusion criteria

(1) Review, case report, in/*ex vitro* and experimental studies, animal studies.
(2) Gestational diabetes.
(3) Taking or using antibiotics or periodontal treatment within 6 months.

#### Data extraction

After study inclusion, two reviewers (ZT and QF) read the full text of the included studies and carried out the data extraction. The characteristics of each study (including patient characteristics, tracking time, measurement data types, etc.), periodontal conditions (including CAL, PD, BOP) and metabolic changes (including glycemic status, HbA1c levels) were extracted. The number of people analyzed, the arithmetic means and the standard deviation of the above data at different follow-up times were also collected. Two reviewers extracted data independently and in duplicate. Disagreements were resolved by discussion with a third reviewer (LH).

### Risk of bias assessment

Two reviewers (ZT and QJ) independently assessed the risk of bias of all included studies using the Cochrane Collaboration tool. Bias can be divided into the following areas: random sequence generation, allocation concealment, blinding of patients, trial personnel, blinding of outcome assessors, incomplete outcome data, selective reporting, and other biases. Different bias situations were divided into the following three categories: 1) High risk (+); 2) low risk (−); 3) unknown (?). Two reviewers discussed and resolved disagreements.

### Statistical analysis

Meta-analysis was performed with Review Manager 5.4.1 (The Nordic Cochrane Centre, The Cochrane Collaboration, Copenhagen, Denmark). A preliminary meta-analysis of eligible RCTs on CAL, PD, BOP and HbA1c levels was performed. Subgroup analyses were performed according to patient characteristics identified in the study. Differences between control and experimental outcomes at different follow-up time were expressed as the weighted mean of consecutive outcomes and 95% confidence intervals. Heterogeneity analysis (Higgins index I^2^) was performed on the mean differences of results at different follow-up time, when I^2^ ≥ 50% using a random model, and I^2^ < 50 a fixed model. Publication bias was assessed by visualization of funnel plots. The quality of the evidence and confidence in the estimates were assessed using the GRADE Working Group criteria.

## Result

**FIGURE 1:**
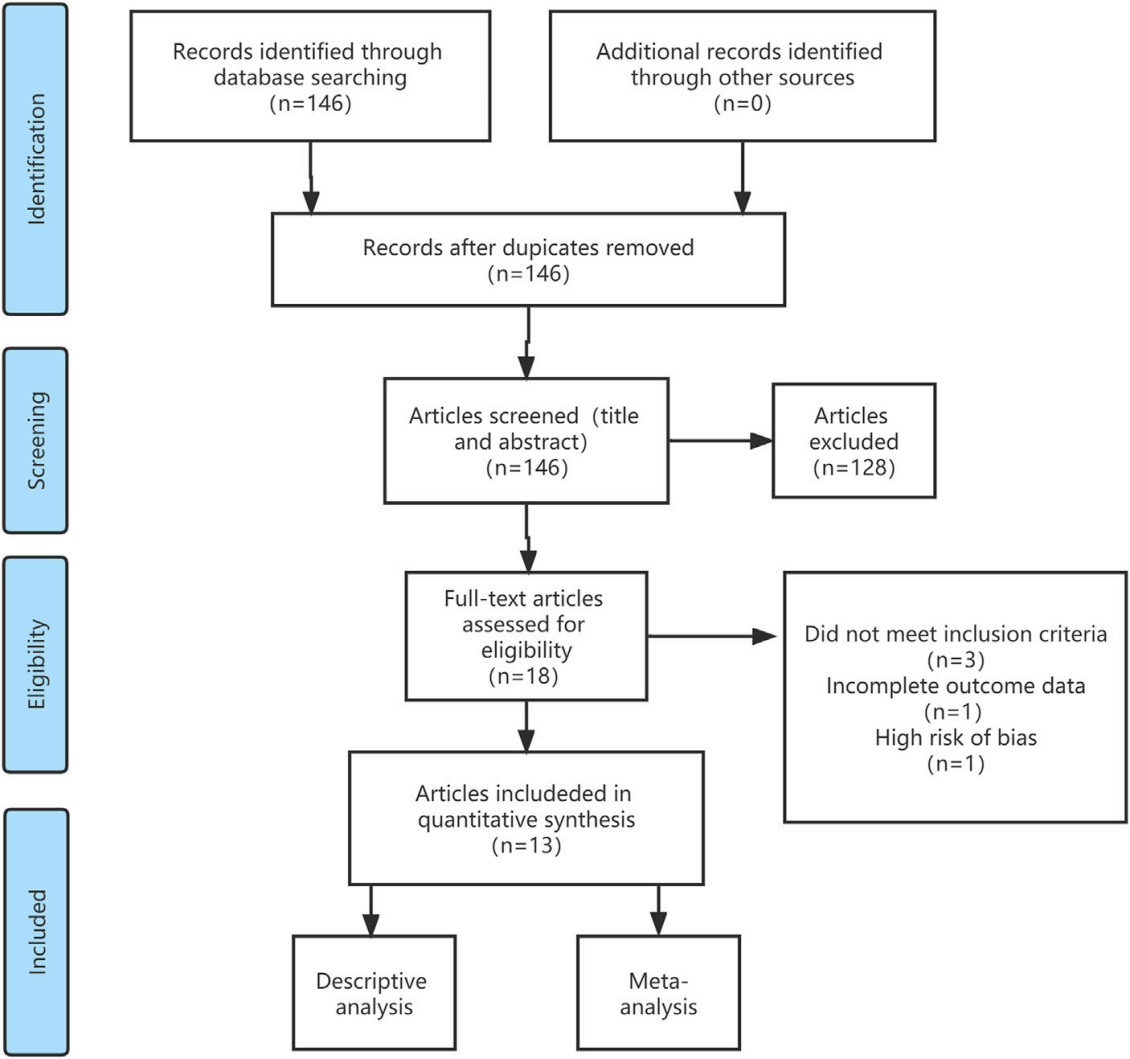
Flow chart of the literature search and inclusion criteria.

A total of 146 studies within the past 10 years were retrieved using the designed search strategy. After excluding duplicate articles and non-English published articles, 13 of them met the inclusion and exclusion criteria. Figure 1 shows the process of identification, screening, eligibility, and inclusion.

**FIGURE 2:**
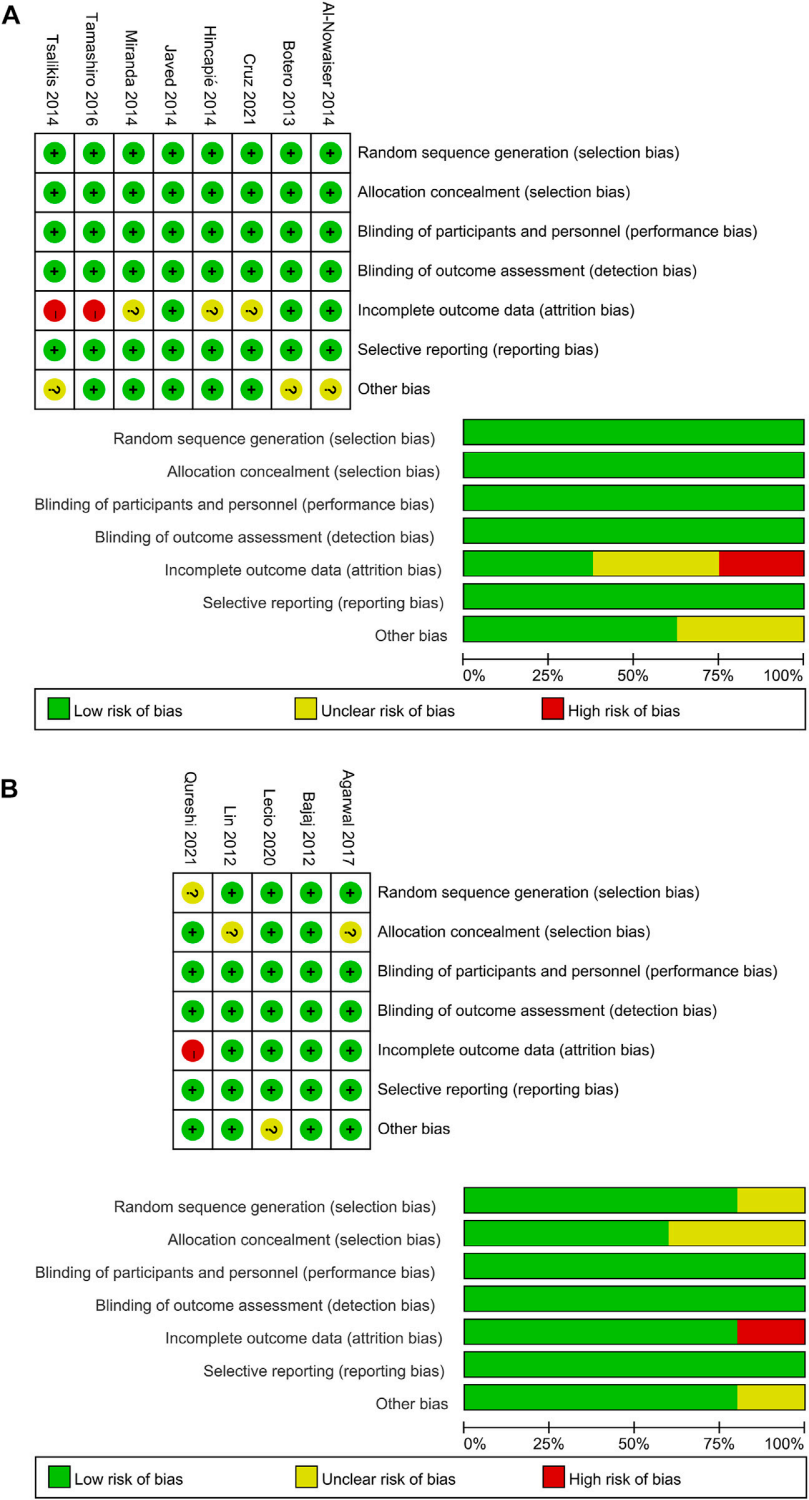
Quality assessment of the included studies using the Cochrane Collaboration tool for assessing risk of bias. **(A)** Systemic antibiotics. **(B)** Topical antibiotics.

Figure 2 shows risk of bias information of all included studies. The overall risk of bias in the 10 studies was low. The remaining three studies were more biased due to the incomplete reporting of results.

The characteristics of the 13 included articles are presented in Tables 1, 2. All of the included studies were RCTs focused on systemic or topical antibiotic usage in diabetic patients with periodontitis. Eight studies focused on the use of systemic antibiotics, while the other five studies on topical antibiotics. Among the studies on systemic antibiotics, five of them included only T2MD participants while the rest included both T1MD and T2MD.

**TABLE 1:** General information provided in the included studies——systemic medication.

Study	Design	Age	Inclusion criteria		Intervention	The mode of administration (dose, time)	Following-up	Assessment
		M/F	HbA1c	PD				
2021 Cruz	RCT	Test: 60.5 ± 7.6 N = 5/10	6.5%–11%	PD ≥ 4 mm	Test: SRP + MTZ + AMX	MTZ 400 mg tid	2 years, 5 years	PD
		Control: 59.6 ± 6.6 N = 2/8			Control: SRP + placebo	AMX 500 mg tid		BOP
						14 days		CAL
								Microorganisms
2016 Tamashiro	RCT	Test: N = 29	≥6.5%	PD ≥ 5 mm	Test: SRP + MTZ + AMX	MTZ 400 mg tid	3 months, 1 year, 2 years	Microorganisms
		Control: N = 27			Control: SPR + placebo	AMX 500 mg tid		
						14 days		
2014 Miranda	RCT	Test: 54.0 ± 8.2 N = 29	6.5%–11%	PD ≥ 4 mm	Test: SRP + MTZ + AMX	MTZ 400 mg tid	3 months, 6 months, 12 months	PD
		Control: 53.7 ± 8.0 N = 27			Control: SPR + placebo	AMX 500 mg tid		BOP
						14 days		CAL
								HbA1c
								Microorganisms
2014 Tsalikis	RCT	Test: 62.9 ± 10 N = 18/13	<7.5%	PD ≥ 5 mm	Test: SRP + Doxy	Doxy 200 mg as loading dose and 100 mg for 20 days	3 months, 6 months	PD
		Control: 57.94 ± 8.22 N = 20/15			Control: SRP			BOP
								CAL
								HbA1c
								Microorganisms
2014 Hincapié	RCT	Test: 55.9 ± 12.6 N = 11/22	NA	PD ≥ 4 mm	Test: SRP + AZM	AZM 500 mg/3 d	3 months, 6 months, 9 months	HbA1c
T1MD + T2MD		Control: 58.2 ± 11.1 N = 10/27			Control: SRP + placebo			Microorganisms
2014 Javed	RCT	Test: 40.7 ± 1.2 N = 33/0	5.7%–6.4%	PD ≥ 5 mm	Test: SRP + Doxy	Doxy 100 mg/d 15 d	3 months	HbA1c
T1MD + T2MD		Control: 44.2 ± 1.4 N = 33/0			Control: SRP			CAL
2014 Al-Nowaiser	RCT	M/F = 47/21 age: 42 ± 6.41	5.8%–10%	PD ≥ 5 mm	Test: SRP + Doxy	Doxy 200 mg as loading dose and 100 mg for 14 days	1 month, 3 months, 6 months	PD
		Test: N = 35			Control: SRP			BOP
		Control: N = 33						CAL
								Microorganisms
2013 Botero	RCT	Test:55.9 ± 12.6 N = 11/22	NA	PD ≥ 5 mm	Test: SRP + AZM	AZM 500 mg/d 3 days	3 months, 6 months, 9 months	PD
T1MD + T2MD		Control:58.2 ± 11.1 N = 10/27			Control: SRP + placebo			BOP
								CAL
								HbA1c

SRP, scaling and root planning; MTZ, Metronidazole; AMX, Amoxicillin Doxy, Doxycycline; AZM, azithromycin.

**TABLE 2:** General information provided in the included studies——topical administration.

Study	Design	Age	Inclusion criteria			Intervention	The mode of administration	Following-up	Assessment
		M/F	HbA1c		PD				
2021 Qureshi	RCT	35–65 y	>6.5%	PD ≥ 4 mm		Test: SRP + MET + OHI	MET 400 mg × 3 10 days + warm salt water 3–5 days	3 months, 6 months	PD
		Test: N = 30/20	<14%			Control: SRP + OHI			BOP
		Control: N = 27/23							CAL
									HbA1c
2020 Lecio	RCT	Test: 58.6 ± 12.4 N = 8/12	NA	PD ≥ 5 mm		Test: FMUD + Doxy	20% Doxy	1 month, 3 months, 6 months	PD
		Control: 53.1 ± 10.2 N = 6/14				Control: FMUD + placebo			BOP
									CAL
									HbA1c
2017 Agarwal	RCT	30–50 year	NA	PD ≥ 5 mm		Test: SRP + AZM	0.5% AZM 0.2 mL	3 months, 6 months, 9 months	PD
		Test: N = 27				Control: SRP + placebo			BOP
		Control: N = 29							CAL
2012 Lin	RCT	Test:56 ± 6 N = 3/11	≥8.5%	PD ≥ 5 mm		Test: SRP + minocycline	2% minocycline gel	3 months, 6 months	PD
		Control:59.0 ± 6.5 N = 5/9				Control: SRP			BOP
									CAL
2012 Bajaj	RCT	Test: N = 29	NA	PD ≥ 5 mm		Test: SRP + clarithromycin	0.5% clarithromycin	1 month, 2 months, 3 months	PD
		Control: N = 27				Control: SRP			BOP
									CAL

**FIGURE 3:**
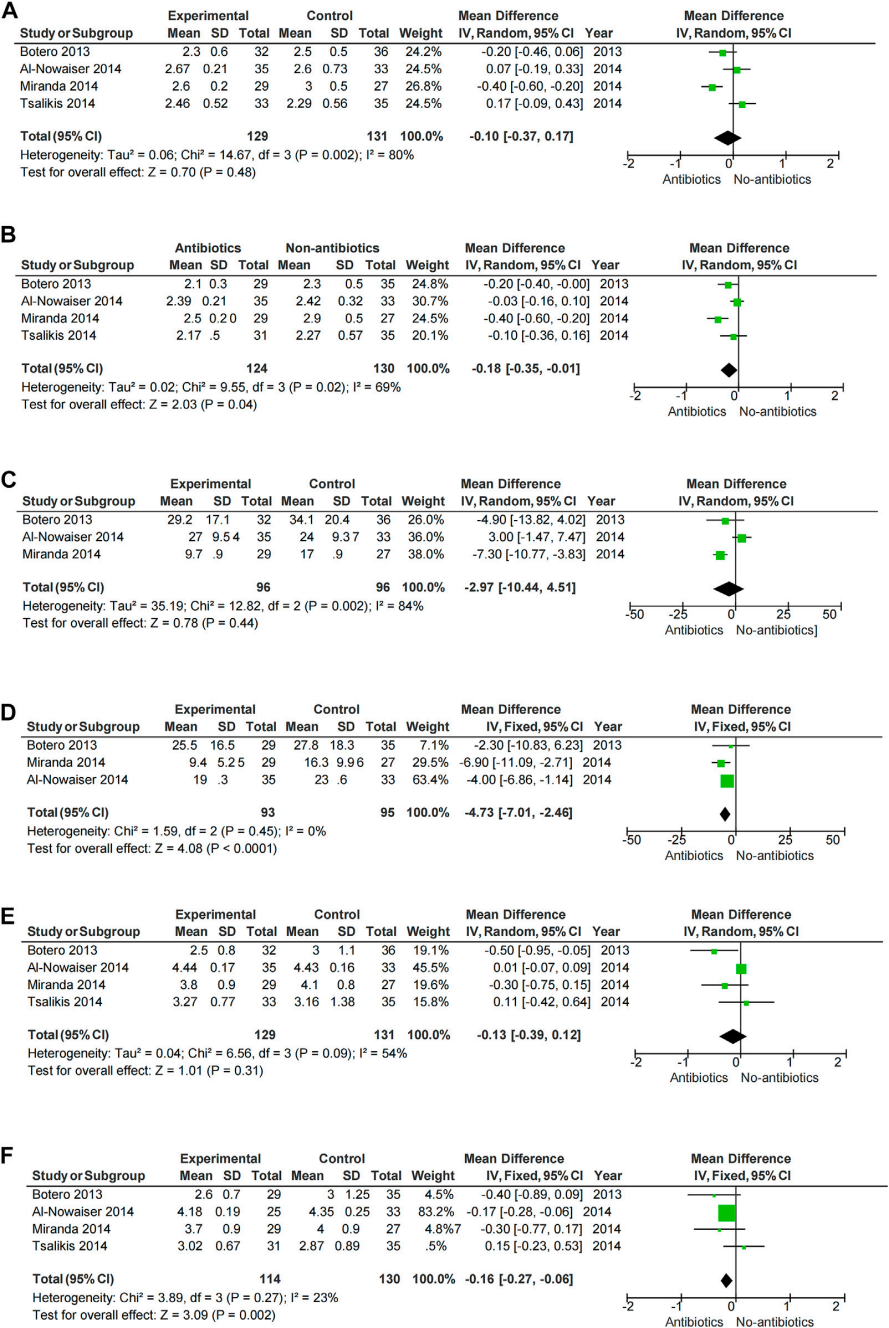
Forest plot of the effect of systemic adjuvant antibiotics on periodontal changes. **(A–B)** Changes in PD at 3 and 6 months. **(C–D)** Changes in BOP at 3 and 6 months. **(E–F)** Changes in CAL at 3 and 6 months.

**FIGURE 4:**
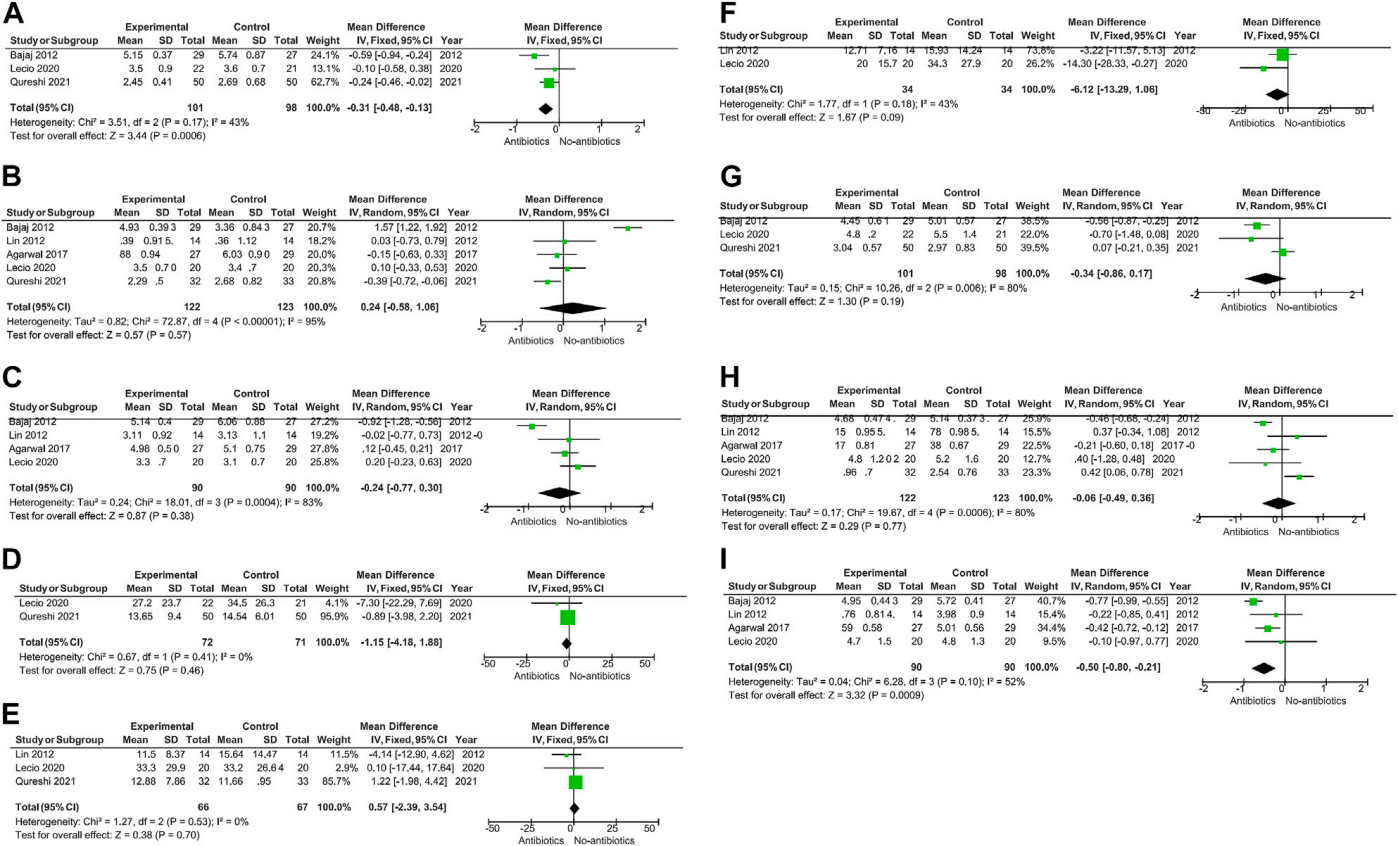
When topical antibiotics were used, there was a significant improvement in PD at 1 month (*p* = 0.0006) and CAL at 6 months (*p* = 0.0009). However, there were no statistically significant differences in the other data.

A meta-analysis was conducted according to the different characteristics of the studies, some of which showed low heterogeneity. The heterogeneity of BOP-6M (I^2^ = 0%) and CAL-6M (I^2^ = 23%) were low in SRP with or without systemic antibiotics (Figures 3D, E). Among studies of topical antibiotics usage, the heterogeneity of PD-1M (I^2^ = 43%) was low (Figure 4A).

**FIGURE 5:**
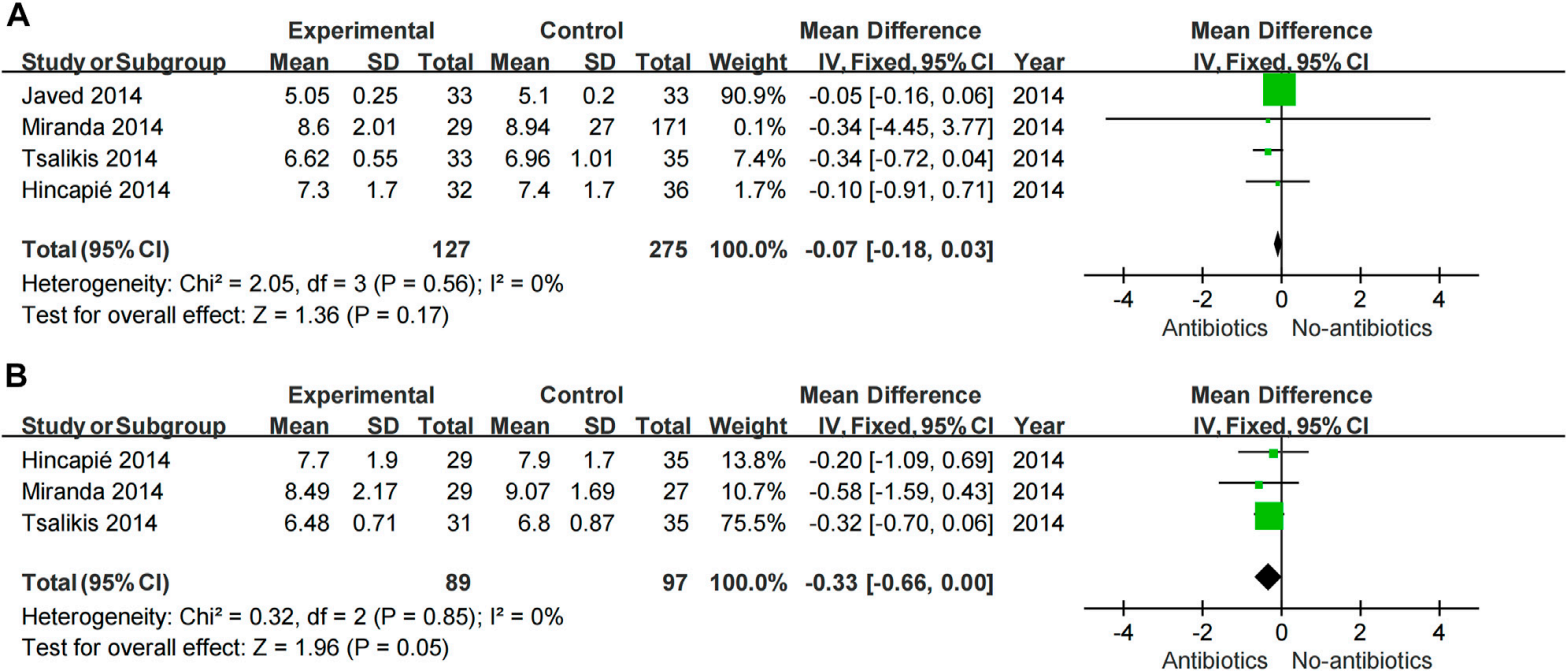
Forest plot of the effect of systemic adjuvant antibiotics on HbA1c. **(A)** Changes in HbA1c at 3 months. **(B)** Changes in HbA1c at 6 months.

**FIGURE 6:**
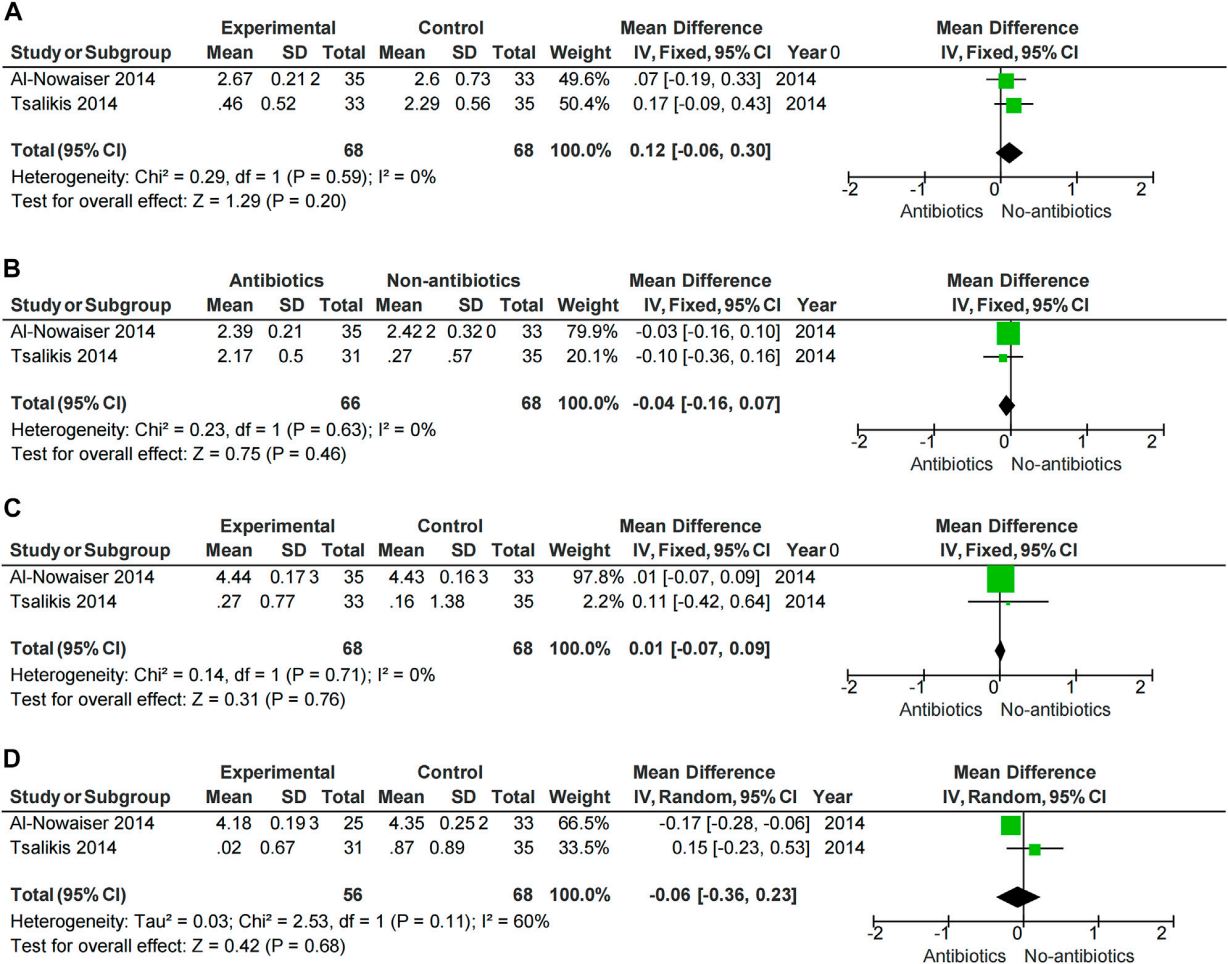
Forest plot of the effect of Doxycycline on periodontal changes. **(A–B)** Changes in PD at 3 and 6 months. **(C–D)** Changes in CAL at 3 and 6 months.

Meta-analysis showed that SRP combined with antibiotics improved PD, BOP, and CAL at 6 months compared with no systemic antibiotics (PD-6M *p* = 0.04, BOP-6M *p* < 0.0001, CAL-6M *p* = 0.002) (Figures 3B, D, F), while there was no significant change at 3 months (PD-3M *p* = 0.48, BOP-3M *p* = 0.44, CAL-3M *p* = 0.31) (Figures 3A, C, E). However, the use of systemic antibiotics on the basis of SRP had no significant effect on HbA1c (HbA1c-3M *p* = 0.17, HbA1c-6M *p* = 0.05) (Figure 5). In three studies (Gaikwad et al., 2013; Al-Nowaiser et al., 2014; Tsalikis et al., 2014), the antibiotics used was doxycycline, and it was found that doxycycline had no significant effect on improvement of PD (PD-3M *p* = 0.20, PD-6M = 0.46) and CAL (CAL-3M *p* = 0.76, CAL-6M *p* = 0.68) at 3 and 6 months (Figure 6).

When topical antibiotics were used, there was a significant improvement in PD at 1 month (*p* = 0.0006) and CAL at 6 months (*p* = 0.0009) (Figures 4A, I).

**FIGURE 7:**
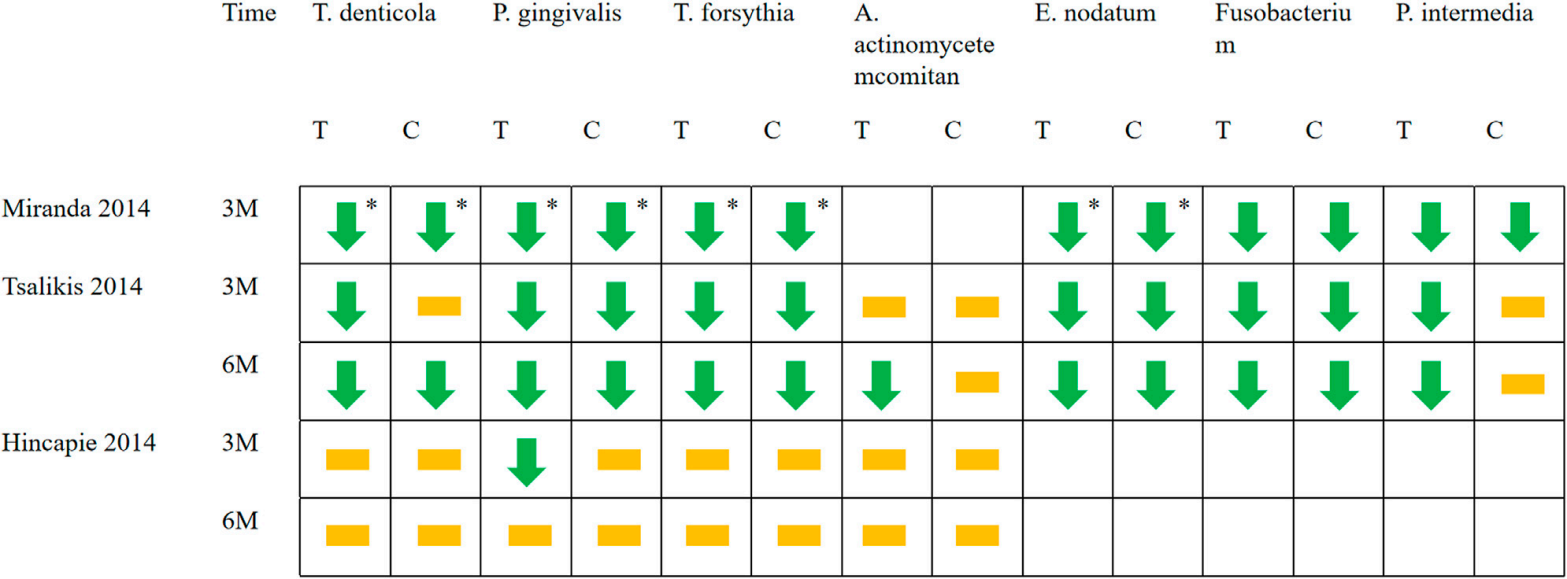
Changes in bacterial species applied with systemic antibiotics. Green arrows represent the decrease of a certain bacteria in this period compared with baseline (*p* < 0.05). The yellow squares represent that compared with the baseline, although there are numerical changes in the number of bacteria, there is no statistical difference. * Represents between-group differences (*p* < 0.05). T represents test group and C represents control group.

**FIGURE 8:**
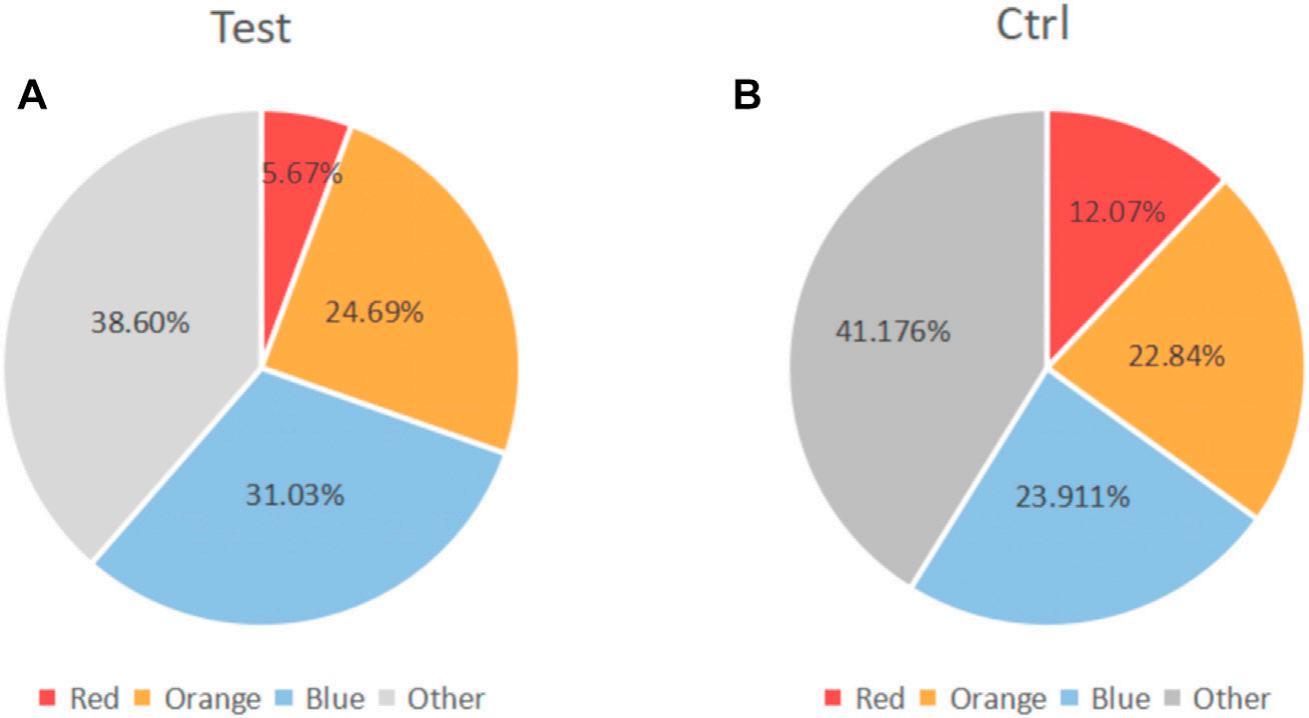
Changes in the proportion of microbiota complexes after adjuvant systemic antibiotics usage. **(A)** Experimental group. **(B)** Control group.Red part: red complex (including *Porphyromonas. Gingivalis, Tannerella forsythia,* and *Treponema denticola*). Orange part: Orange complex. Blue part, blue complex. Grey part, Other complex.

Some of included studies (Hincapié et al., 2014; Miranda et al., 2014; Tsalikis et al., 2014) performed statistical analyses of bacterial species. It was found that compared with baseline, P.g, T.f, T.d, E.n, *Fusobacterium* and P.i all decreased to a certain extent. Only the Miranda 2014 study found between-group differences in bacterial (P.g, T.f, T.d, E.n) reduction. (Figure 7). Two studies (Tamashiro et al., 2016; Cruz et al., 2021) performed statistical analyses of periodontal biocomplexes and found that complexes closely related to periodontal disease were numerically reduced after adjunctive antibiotic use (Figure 8).

## Discussion

Periodontitis is an infectious disease, therefore, the success of periodontal treatment depends on anti-infective treatment, the main purpose of which is to eliminate the plaque and pathogenic microorganisms on the tooth surface. Currently, it is generally accepted that mechanical manipulation can effectively remove supragingival and subgingival calculus and plaque, thereby maintaining the long-term stability of periodontal tissue (Drisko, 2001). Diabetes is currently recognized as one of the risk factors for periodontitis, and people with diabetes are more likely to develop more severe periodontitis. Only mechanical removal of calculus and plaque was found not effective in diabetic patients with severe periodontal destruction (Kocher et al., 2018). Therefore, many studies explored the effect of adjunctive antibiotics usage on diabetic patients with periodontitis, so as to explore whether antibiotic-assisted periodontal mechanical treatment is effective. However, since microbial resistance and systemic flora were both affected by antibiotics, an assessment between efficacy and risk is important (Pretzl et al., 2019). Considering the improvement of patients’ periodontal condition and drug side effects, whether to use antibiotics to assist periodontal mechanical therapy is still controversial.

Periodontitis is characterized by loss of periodontal attachment and gingival inflammation, so PD, BOP, and CAL become the main criteria for periodontitis evaluation. In our study, results of clinical parameters (PD, BOP, CAL, and HbA1c) were mainly collected at 3 months and 6 months. We found that SRP combined with the systemic administration of antibiotics had effect on the improvement of periodontal condition in the long term (6 months). A 2018 meta-analysis (Souto et al., 2018) also found that adjunctive use of systemic antibiotics had an additional benefit in PD improvement (0.14 mm reduction in PD) compared with patients treated with non-surgical periodontal therapy alone. Rovai et al. (2016) found that systemic adjunctive antibiotics were beneficial in reduce of CAL and PD, especially in deep periodontal pockets. However, we found no significant effect in the short term (3 months) when SRP and systemic antibiotics usage were also both applied. This suggested in the short-term periodontal condition was improved mainly by mechanical means, and systemic antibiotics usage benefits periodontal health in a long run. For most periodontitis pathogen doxycycline is a common antimicrobial agent. However, subgroup analysis showed no significant periodontal benefit with adjunctive use of doxycycline, and thus does not support the use of doxycycline in diabetic patients with periodontitis. In addition, the results of Lira Junior et al. (2017) showed that the adjunctive use of systemic antibiotics in the periodontal treatment of diabetic patients had no statistically significant improvement in HbA1c (HbA1c-3M *p* = 0.17, HbA1c-6M *p* = 0.05) (Figure 5). Evidence for the effect of non-periodontal surgery on glycemic changes is also limited, and Engebretson et al. (2013) found no significant improvement in glycemia with non-periodontal surgery. Therefore, the periodontist should weigh the risks and benefits when using systemic antibiotics.

Socransky et al. (1998) observed that the subgingival bacteria aggregated with certain rules. According to their aggregation characteristics and their relationship with periodontal conditions, the subgingival bacteria were divided into six major microbial complexes, represented by red, orange, yellow, green, purple, and blue, respectively. The red compound concludes the flora that is closely related to periodontitis, including: *Porphyromonas. Gingivalis, Tannerella forsythia* and *Treponema denticola*. However, we only included two studies that investigated changes in microbial complexes. We found that antibiotics can numerically decrease the number of microorganisms closely associated with periodontal diseases. Systemic antibiotic therapy appears to improve periodontal status by altering the composition of periodontal plaque microbes. We need more relevant studies to explore the changes of microorganisms in the systemic adjuvant antibiotics usage.

In the study of topical antibiotics usage, we found that short-term (3 months) use of antibiotics was beneficial for PD improvement. This may be related to the fact that effective concentrations of drugs in the gingival sulcus within a relatively short time can be more easily achieved by topical administration and thus change the composition of subgingival organisms. Moreover, topical administration has fewer adverse drug effects.

The early stages of periodontitis with diabetes were caused by inflammation-induced death of periodontal endothelial and other cells (Graves et al., 2020). And the inflammatory factors produced in periodontal disease may adversely affect the glycemic control of diabetes. Therefore, it has been suggested that there may be a bidirectional relationship between periodontal status and glycemic level control (Sandberg et al., 2000; Simpson et al., 2015). Although our results found no effect of periodontal treatment with or without adjuvant antibiotics on HbA1c, some researchers suggested that periodontal treatment can reduce HbA1c (Simpson et al., 2010; Simpson et al., 2015; Madianos and Koromantzos, 2018), which may be related to the reduction of inflammatory factors such as TNF-α, C-reactive protein (CRP) and oxidative stress markers *in vivo* after periodontal treatment. These inflammatory factors were at elevated levels in the circulation of both diabetic and periodontitis patients (Polak and Shapira, 2018; Preshaw and Bissett, 2019). This may also point to a link between the two.

In the studies we included, none reported adverse effects from antibiotic use, only mild side effects such as gastrointestinal distress were observed. In addition, the metabolic level and smoking status of the patients were also different, but no statistical heterogeneity difference was found. We need more high-quality clinical trials to understand the detailed effects of antibiotic combined periodontitis treatment for diabetic patients with periodontitis.

## Conclusion

In diabetic patients with periodontitis, systemic antibiotics had significant improvement in PD and CAL in long-term. Topical antibiotics were also beneficial for PD improvement. However, no significant benefits in periodontal status were observed in short-term systemic antibiotics usage. Improvement in periodontal status was found. Periodontal treatment with or without adjuvant antibiotics showed no effect on HbA1c regulation. Clinical evaluation should be based on the benefits and risks of using antibiotics with periodontal treatment for diabetic patients with periodontitis.

## Data Availability

The original contributions presented in the study are included in the article/supplementary material, further inquiries can be directed to the corresponding author.
